# Gut–lung axis in radiation-induced lung injury: mechanisms and interventions

**DOI:** 10.3389/fimmu.2026.1806833

**Published:** 2026-07-02

**Authors:** Ping Zhou, Xiao Jiang, Haiyan Zhang, Shizheng Jiang, Xiaotao Zhang, Chengtai Ma, Xiaoshuai Bai

**Affiliations:** 1 Department of Oncology, Qingdao Central Hospital, University of Health and Rehabilitation Sciences (Qingdao Central Hospital), Qingdao, China; 2 School of Clinical Medicine, Shandong Second Medical University, Weifang, Shandong, China; 3 Qingdao Traditional Chinese Medicine Hospital, Qingdao Hiser Hospital Affiliated of Qingdao University, Qingdao, Shandong, China; 4 Department of Emergency Internal Medicine, the Affiliated Hospital of Qingdao University, Qingdao, China

**Keywords:** gut–lung axis dysregulation, immune pathways, microbial dysbiosis, microbiome-host interactions, pulmonary fibrosis, radiation-induced lung injury

## Abstract

Radiation-induced lung injury (RILI) constrains thoracic radiotherapy dosing and includes acute radiation pneumonitis (RP) and chronic radiation-induced pulmonary fibrosis (RPF). This narrative review explores the gut–lung microbiota axis in RILI, synthesizing evidence from preclinical models, clinical cohorts (N = 52–89), and randomized controlled trials (RCTs). Radiotherapy induces gut dysbiosis, barrier breakdown, and metabolite changes [e.g., short-chain fatty acid (SCFA) and desaminotyrosine (DAT) depletion], promoting inflammation and fibrosis via pathways such as Toll-like receptor 4/nuclear factor kappa B (TLR4/NF-κB), TGF-β/Smad, sphingosine-1-phosphate (S1P)–S1PR, and cGAS–STING in animal studies. Inter-species microbial variations hinder translation, while lung microbiota shifts remain nascent. In non-small cell lung cancer cohorts, lower gut microbiota stability (a marker of dysbiosis) is associated with an increased risk of grade ≥2 RP (multivariable-adjusted models, p < 0.05), with higher baseline *Faecalibacterium* abundance conferring protection; however, causality remains unproven due to antibiotic confounding. Mechanisms involve lipopolysaccharide (LPS) translocation, interleukin 25 (IL-25)/S1P-driven type 2 innate lymphoid cell (ILC2) migration, regulatory T cell/T helper 17 cell (Treg/Th17) imbalance, and extracellular vesicle (EV) signaling, with biomarkers such as 16S rRNA sequencing and EV-miRNAs (e.g., miR-486-5p). Artificial intelligence models predict RP with 75% accuracy. Phase-specific interventions, such as pre-radiotherapy gut microbiota monitoring, intra-radiotherapy SCFA supplementation, subacute DAT modulation, and RPF-targeted EV therapies, have been explored in preliminary pilot studies [for example, one small study reported approximately 12% FEV1 improvement following fecal microbiota transplantation (FMT)]. Future large-scale, stratified RCTs that properly account for antibiotics, chemotherapy, and immunotherapy are required to establish causality beyond the current largely associative clinical evidence. The integration of immunotherapy and proton therapy in such trials may help clarify gut–lung interactions, including any microbiota-preserving effects of proton therapy; the role of the lung microbiota in fibrosis remains preliminary.

## 1 Introduction

Radiation-induced lung injury (RILI) is a dose-limiting complication of thoracic radiotherapy (RT) for intrathoracic and adjacent malignancies, encompassing acute radiation pneumonitis (RP) (typically 1–6 months post-RT) and chronic radiation-induced pulmonary fibrosis (RPF) (>6 months) (1). Clinically, RILI constrains dose escalation and tumor control probability (TCP) while impairing quality of life through dyspnea, non-productive cough, low-grade fever, hypoxemia, and fatigue; severe cases [grade 3–5 Common Terminology Criteria for Adverse Events (CTCAE)] may require oxygen therapy and hospitalization or can result in death (2). Modern thoracic RT modalities include photon-based techniques—three-dimensional conformal RT (3D-CRT), intensity-modulated RT (IMRT)/volumetric modulated arc therapy (VMAT)—and advanced approaches such as stereotactic body RT (SBRT) for early-stage or oligometastatic disease and proton beam therapy (PBT). Photon beams deliver energy along their entire path with an exit dose beyond the target, increasing integral lung dose; IMRT/VMAT improves conformity and reduces high-dose lung volume (e.g., V20) compared with older 3D-CRT. SBRT delivers ablative doses (e.g., 48–60 Gy in 3–5 fractions) in 1–2 weeks with steep gradients, minimizing irradiated lung volume. PBT exploits the Bragg peak to deposit most energy at the target depth with no exit dose, sparing the distal lung, heart, and esophagus—potentially reducing the risk of developing RILI compared with photon-based RT in dosimetric studies and early clinical comparisons, especially when combined with immunotherapy. Treatment duration varies by intent and technique. For locally advanced non-small cell lung cancer (LA-NSCLC) or esophageal cancer, definitive concurrent chemoradiotherapy (CRT) typically delivers 50–60 Gy in 25–30 fractions (1.8–2 Gy/fx) over 5–6 weeks; SBRT shortens this to 1–2 weeks. Postoperative or palliative regimens may use shorter courses (e.g., 30–40 Gy/10–15 fx). Esophageal protocols often include prophylactic nodal irradiation, increasing lung exposure, while NSCLC plans prioritize involved-field irradiation with modern image guidance. Incidence and severity differ markedly by cancer type, modality, and concurrent therapies. In LA-NSCLC (the most common thoracic RT setting), symptomatic (grade ≥2) RP occurs in 10%–30% of patients, with grade 3–5 in 5%–15%; radiographic changes are near-universal (>80%). Concurrent chemotherapy (especially gemcitabine or taxanes) and baseline interstitial lung disease (ILD) elevate risk; V20 > 20%–25% or mean lung dose (MLD) > 18–20 Gy strongly predicts events. In esophageal squamous cell carcinoma (ESCC), RP incidence is ~22% (grade ≥4 ~ 1.5%), driven by larger mediastinal fields. Breast cancer RT (tangential or nodal fields) shows lower symptomatic RP (2%–19% grade 1–2; rarely >grade 2), although radiologic fibrosis is common (~90% at 12 months). Hematologic malignancies (historical mantle fields) or SBRT cohorts report <10% grade ≥2 RP with modern constraints. Proton therapy cohorts consistently show 8%–12% grade ≥2 RP versus 15%–25% with photons in comparable thoracic volumes. These complications remain clinically relevant: severe RP limits TCP by 10%–20% in high-risk cases, reduces 3-year Overall Survival (OS), and affects 15%–40% of patients overall, modulated by smoking status, comorbidities, and immunotherapy (e.g., consolidation durvalumab after CRT increases pneumonitis overlap) (3). Glucocorticoids remain first-line for symptomatic RP, but antifibrotics offer limited benefit for established RPF (4). Recent data, including the 2025 models of uranium exposure and protozoal roles in the gut–lung axis (5), reveal bidirectional immune-metabolic shifts, with lung injury affecting gut microbiota and metabolites (6). This review synthesizes recent 2024–2025 studies linking the gut–lung axis to respiratory diseases and radiotherapy outcomes, which is relevant for microbiologists, oncologists, and clinicians. Preclinical models and small observational cohorts have reported associations between microbiome modulation and reduced RILI severity in vulnerable patients; however, lung microbiome evidence remains an emerging area of study, and any quantitative clinical benefit estimates require confirmation through large-scale randomized controlled trials (RCTs).

The gut–lung axis forms a bidirectional link between the intestinal microbiota and lung homeostasis, playing a role in the progression of respiratory disorders (7). It is driven by microbial metabolites [e.g., short-chain fatty acids (SCFAs) such as butyrate and propionate]., immune cell trafficking [e.g., type 2 innate lymphoid cells (ILC2s) and regulatory T cells (Tregs)]., and pathways regulating lung immunity and barriers (5, 8). In radiotherapy, gut dysbiosis and barrier dysfunction impair SCFA production and promote endotoxin leakage, altering macrophage polarization, epithelial repair, and inflammasome activity via G protein-coupled receptors 41/43 (GPR41/43)–histone deacetylase (HDAC) and Toll-like receptor 4 (TLR4)/MyD88-NLRP3 routes (6). Furthermore, interleukin 25/sphingosine-1-phosphate (IL-25/S1P)-guided ILC2 migration, Treg/T helper 17 cell (Th17) imbalance, and cGAS–STING/TGF-β/Smad crosstalk drive RP to RPF progression (9–11). Extracellular vesicle (EV)-associated microRNAs (miRNAs) and lipids increase endothelial permeability and matrix buildup amid radiation-triggered reactive oxygen species (ROS) and DNA damage (12–14).

### 1.1 Aims and scope

This review sought to 1) synthesize preclinical and clinical data on gut–lung axis mechanisms in RILI; 2) assess the correlation between gut microbiota dysbiosis and RILI progression, noting nascent lung microbiota roles; and 3) suggest phased biomarkers and interventions for clinical use. The core questions address how radiotherapy-driven gut dysbiosis may contribute to lung inflammation and fibrosis through specific pathways in preclinical models, the strengths and limitations of largely associative clinical evidence (confounded by antibiotics, chemotherapy, and immunotherapy), and the potential of microbiome strategies such as fecal microbiota transplantation (FMT) and SCFA supplementation in mitigating RILI across phases, pending validation in adequately powered RCTs. We connect gut–lung elements—metabolites, immune cells, and EVs—with pathways [TLR4/nuclear factor kappa B (NF-κB), TGF-β/Smad, S1P–S1PR, and cGAS–STING]. Strategies include time-based biomarkers and interventions for pre-radiotherapy, the treatment period, and the management of subacute RP and RPF, aiding precise care.

### 1.2 Methods

This narrative review used a structured search to ensure breadth and minimize bias. PubMed, Embase, Web of Science, and Scopus were queried for literature spanning January 2010 to September 2025 based on the following terms: “gut-lung axis” OR “microbiota axis” AND “radiation-induced lung injury” OR “RILI” OR “radiation pneumonitis” OR “pulmonary fibrosis” AND (“mechanisms” OR “interventions” OR “dysbiosis” OR “SCFAs” OR “DAT” OR “FMT”). Eligibility criteria were restricted to English-language peer-reviewed papers on preclinical models, clinical cohorts, RCTs, and reviews concerning the gut–lung axis in RILI. Eligibility criteria excluded studies that were not written in English, case reports (N < 10), and unrelated articles (e.g., non-radiation-induced lung injuries). From 450 abstracts and 200 full texts, 99 references were chosen. To counter publication bias, negative/null findings (e.g., variable FMT results) were included, and a critical appraisal of evidence strength was performed based on study design, sample size, confounding factors, consistency across studies, and applicability to human RILI. Databases were chosen for their broad biomedical scope, targeting oncology and microbiology. The period covered post-2010 microbiome sequencing and radiation advances, excluding earlier studies. Terms balanced detail (e.g., RILI mechanisms) and breadth for new areas such as SCFAs, desaminotyrosine (DAT), and FMT. Reference snowballing reduced selection bias. This approach follows narrative review standards, yielding targeted synthesis. Evidence limitations are discussed narratively in the relevant sections and summarized in **Table 1**.

**Table 1:** Summary of evidence, strengths, and limitations in gut–lung axis and RILI.

Key association	Evidence type	Summary of findings	Evidence strength assessment	Rationale for evidence assessment	Supporting references
Gut dysbiosis and increased RP risk	Observational cohorts (e.g., NSCLC, N = 45–89); meta-analyses; preclinical models	Dysbiosis (e.g., reduced α-diversity, Proteobacteria overgrowth, *Bifidobacterium*/*Lactobacillus* decline) associated with >30% increased RP risk (OR = 2.8–3.1, p < 0.05). Mechanisms: LPS translocation, SCFA depletion, TLR4/NF-κB activation. Consistent across thoracic cancers, but confounded by antibiotics/chemotherapy.	Limited observational evidence	Observational data limited by high risk of bias from confounders (antibiotics, chemotherapy, and immunotherapy; reverse causation), inconsistency (I^2^ > 50% heterogeneity), and imprecision (small N = 45–89). Publication bias favors positive findings. No large randomized trials available. Preclinical models show stronger mechanistic support but face translation challenges.	Xi et al. (2025) (15), Zeng et al. (2023) (24), Charalambous et al. (2025) (108), Wang et al. (2025) (4) meta-analysis, Enaud et al. (2020) (16)
FMT efficacy in mitigating RILI	RCTs/pilot studies (e.g., N = 5–120); preclinical (murine models); case series	FMT improves lung function (e.g., 12% FEV1 gain), reduces inflammation/fibrosis, and restores microbiota (e.g., via PGF2α/MAPK/NF-κB). Safe in Carbapenem-resistant Enterobacteriaceae (CRE); preclinical shows reduced RP severity. Mixed outcomes due to donor variability. Potential 15%–25% RP reduction in high risk.	Limited to moderate (emerging but preliminary)	Small-scale RCTs and pilot studies are limited by sample size, lack of blinding in some studies, indirectness (mostly preclinical data extrapolated to RILI), and imprecision due to donor variability and lack of stratification for key confounders. Plausible mechanisms exist, but confirmatory large RCTs are needed.	Nie et al. (2020) (17), Gurczynski et al. (2023) (18), Wekking et al. (2025) (19), Chen et al. (2021) (20), Ding et al. (2020), Li et al. (2025) (21)

RILI, radiation-induced lung injury; RP, radiation pneumonitis; NSCLC, non-small cell lung cancer; LPS, lipopolysaccharide; SCFA, short-chain fatty acid; FMT, fecal microbiota transplantation; RCTs, randomized controlled trials; CRE, Carbapenem-resistant Enterobacteriaceae.

## 2 Gut–lung microbiota axis: fundamental physiology and immune pathways

### 2.1 Gut–lung microbiota axis: fundamental physiology, host interactions, and distal signal transduction mechanisms

The gut–lung axis forms a bidirectional network that supports host homeostasis via microbial metabolites, immune cell movement, and signaling pathways, with the intestinal microbiota shaping pulmonary immune tolerance (22). Key immune cell populations bridging the gut and lung include ILC2, Tregs, Th17 cells, and macrophages. Under homeostatic conditions, ILC2s are relatively scarce in both lung and gut mucosal tissues yet serve as the dominant innate lymphoid population in the lung; they act as rapid first responders that produce type 2 cytokines (IL-5, IL-13, and IL-9) and amphiregulin to regulate type 2 immunity against parasites and allergens, promote epithelial repair, and maintain barrier integrity. Tregs maintain immune tolerance and suppress excessive inflammation through IL-10 and TGF-β secretion, while Th17 cells promote mucosal barrier defense and neutrophil recruitment via IL-17A/IL-22, but an imbalance can drive pathogenic inflammation. Macrophages exhibit plasticity, polarizing to proinflammatory M1 [via TLR4/NF-κB, releasing tumor necrosis factor alpha (TNF-α)/interleukin 6 (IL-6)/ROS] or anti-inflammatory/pro-repair M2 phenotypes (via GPR41/43 and HDAC inhibition), thereby linking microbial signals to tissue homeostasis or fibrosis. Dendritic cells (DCs) sample microbial antigens and orchestrate ILC2 migration and T-cell priming across the gut–lung axis. These populations are modulated by gut-derived metabolites (e.g., SCFAs promoting Treg differentiation and M2 polarization) and enable distal signal transduction, setting the stage for dysbiosis-driven pathology in RILI. The vast majority (90%) of the gut microbiota consists of the bacterial phyla Bacteroidetes/Bacteroidota, Firmicutes/Bacillota, and Proteobacteria. Strains such as *Clostridium orbiscindens* produce DAT from flavonoids, while *Bacteroides* species convert dietary fibers to SCFAs such as butyrate and propionate, bolstering epithelial barriers. By contrast, the lung microbiota is sparser—10 to 100 bacteria per 1,000 cells—with overlapping phyla but lower microbial diversity influenced by age and diet. Genera such as *Streptococcus* and *Prevotella* modulate immunity through horizontal gene transfer and quorum sensing. Gut microbiota disruptions can exacerbate lung inflammation systemically, although lung-to-gut feedback is limited. In RILI, radiotherapy triggers gut dysbiosis, promoting the growth of Proteobacteria such as *Escherichia coli* and triggering the release of lipopolysaccharide (LPS), which intensifies inflammation, oxidative damage, and fibrosis, as shown in mouse models using FMT (16, 23). The gut mycobiome modulates immunity, with mounting data on lung mycobiome roles. Radiation shifts fungal profiles, favoring pathobionts such as *Candida*, which promote interleukin 17 (IL-17)/Th17 responses, mirroring idiopathic pulmonary fibrosis (IPF) patterns (11). Gut mycobiome dysbiosis is linked to poorer lung function in chronic conditions, similar to radiation-induced fungal overgrowth, such as *Aspergillus* proliferation (12). The virome influences immunity via bacteriophages; in RILI, gut virome changes weaken antiviral defenses, impairing type I interferon (IFN-I) signaling and prolonging fibrosis, similar to IPF (2, 3). Microbial metabolites enhance responses in influenza models, although lung virome data remain preliminary (13). Microbial diversity is key to homeostasis—high-diversity communities rich in *Lactobacillus rhamnosus* and *Bifidobacterium longum* promote tolerance through quorum-sensing metabolites and curb proinflammatory cytokines, while low-diversity communities raise disease risk. Firmicutes/Bacillota-to-Bacteroidetes/Bacteroidota imbalances trigger IL-33, activating ILC2s and amplifying responses (24, 25). Protozoal factors in the gut–lung axis are an emerging research area and are addressed in detail in the Future Perspectives section. Antibiotics disrupt microbial diversity, favoring Proteobacteria such as *Klebsiella pneumoniae* and causing epithelial harm via biofilms. SCFAs strengthen tight junctions and block pathogens. Proteobacteria have dual roles: commensals aid vitamin production, but pathobionts drive TLR4-mediated inflammation in dysbiosis, according to gnotobiotic research (26, 27). Broader reviews on gut–airway interactions underscore the contributions of fungal and viral communities to both homeostasis and disease pathogenesis (19), as well as the role of the lung microbiome in infectious diseases. Incorporating protozoal components may provide a more comprehensive understanding of radiation effects across different microbial kingdoms (bacteria, fungi, viruses, and protozoa).

Microbial metabolites drive these links in various ways. SCFAs from Bacteroidetes/Bacteroidota and Firmicutes/Bacillota exert anti-inflammatory actions via GPR41/43 and HDAC inhibition, fostering M2 macrophage polarization, Treg differentiation, and NF-κB suppression to downregulate the expression of TNF-α and IL-6 (28, 29). In RILI, low levels of SCFAs decrease oxidative stress, but high levels may promote fibrosis; preclinical data show post-radiotherapy declines (e.g., >50% butyrate loss), which impair tolerance, reduce Tregs, and raise Th17 cells, worsening inflammation and RP. DAT from *C. orbiscindens* boosts IFN-I signaling, curbing apoptosis and cytokine storms, showing promise as an antifibrotic agent (30, 31). Multi-omics reveal amino acid and pyrimidine pathway shifts: thoracic radiation reduces fecal l-histidine and inosine monophosphate, with FMT or l-histidine restoring equilibrium and mitigating inflammation and fibrosis.

**Figure 1:**
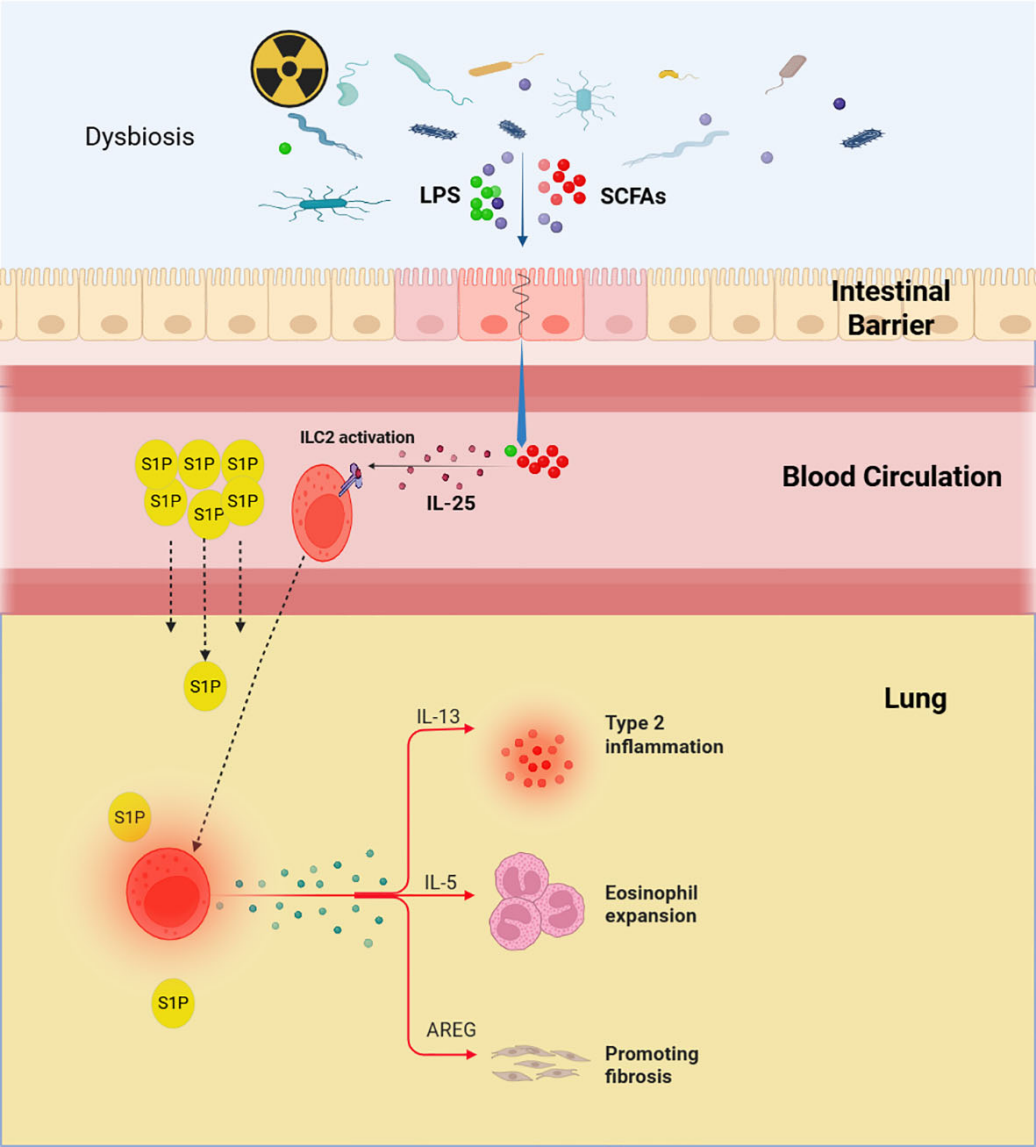
Schematic illustration of the gut–lung axis in radiation-induced pneumonitis: ILC2 activation and migration. Radiation disrupts the intestinal barrier, causing gut dysbiosis, bacterial translocation, and metabolite shifts (e.g., SCFAs), enhancing IL-25 production and inflammatory signals along the gut–lung axis. IL-25 activates ILC2s via IL-17RB, upregulating CCR9 and S1PR1 for S1P-guided lung migration, per mouse models. In the lungs, ILC2s release IL-5, IL-13, and amphiregulin (AREG), promoting eosinophil recruitment, type 2 inflammation, repair, and fibrosis in RP, linked to progression in studies. S1P inhibitors (e.g., FTY720) block influx; SCFA-producing probiotics (e.g., *Lactobacillus*) or FMT restore homeostasis, normalize migration, and mitigate fibrosis in models. SCFAs, short-chain fatty acids; RP, radiation pneumonitis; FMT, fecal microbiota transplantation.

Mucosal barriers support these metabolites to sustain defense. Gut mucin 2 (Muc2) blocks microbial entry, while lung mucins MUC5AC and MUC5B limit pathogen binding. Secretory immunoglobulin A (sIgA) neutralizes antigens and maintains tolerance. Radiation weakens barriers, aiding translocation, but probiotics and high-fiber diets rebuild sIgA and mitigate RILI (32, 33) (**Figure 1**).

## 3 Radiation-induced microbiota dysbiosis and its association with lung injury

Radiotherapy for thoracic tumors disrupts gut microbial balance while targeting cancer cells, thereby contributing to RILI. RILI encompasses acute RP and chronic RPF, with the gut–lung microbiota axis connecting systemic and local responses to drive fibrotic changes. The following sections detail the effects of intestinal and pulmonary dysbiosis, with stronger evidence for gut microbiota changes and emerging data on the lung microbiota.

### 3.1 Effects of radiotherapy on intestinal microbiota

Radiotherapy alters intestinal microbiota equilibrium, reducing diversity and restructuring communities (34). In a longitudinal NSCLC study (N = 52), lower intra-patient gut microbiota stability during CRT was independently associated with higher risk of grade ≥2 RP (multivariable models, p < 0.05), with baseline *Faecalibacterium* enrichment identified as a protective factor; higher stability correlated with reduced RP incidence (15). A separate 2024 cohort (N = 45) linked the proliferation of Proteobacteria and the reduction of *Bifidobacterium* to RP severity, while a 2025 study (N = 89) reported their association with progression [hazard ratio (HR), 1.5]. However, these associations remain correlative and are heavily confounded by concurrent antibiotics, chemotherapy, and immunotherapy; causality cannot be inferred from current observational data alone. Cross-disease parallels, such as shared Proteobacteria and SCFA depletion in LPS/TLR4 signaling observed in chronic obstructive pulmonary disease, asthma, and COVID-19, mirror these patterns but do not establish causality in RILI. Observational cohort evidence is limited by the high risk of bias from confounding factors such as concurrent antibiotics and chemotherapy, potential reverse causation, inconsistency due to patient heterogeneity (I^2^ > 50% in related analyses), and imprecision from small sample sizes (N = 45–89). Preclinical mechanistic studies have provided stronger causal insights into animal models but have faced challenges in translation due to indirectness and species-specific differences in microbiota composition. Central mechanisms include radiation damage to tight junctions such as occludin and zonula occludens-1 (ZO-1), increasing permeability, LPS translocation, and dose-related systemic inflammation with heightened infection risk (35–37).

### 3.2 Microbiota dysbiosis and RILI mechanisms

**Figure 2:**
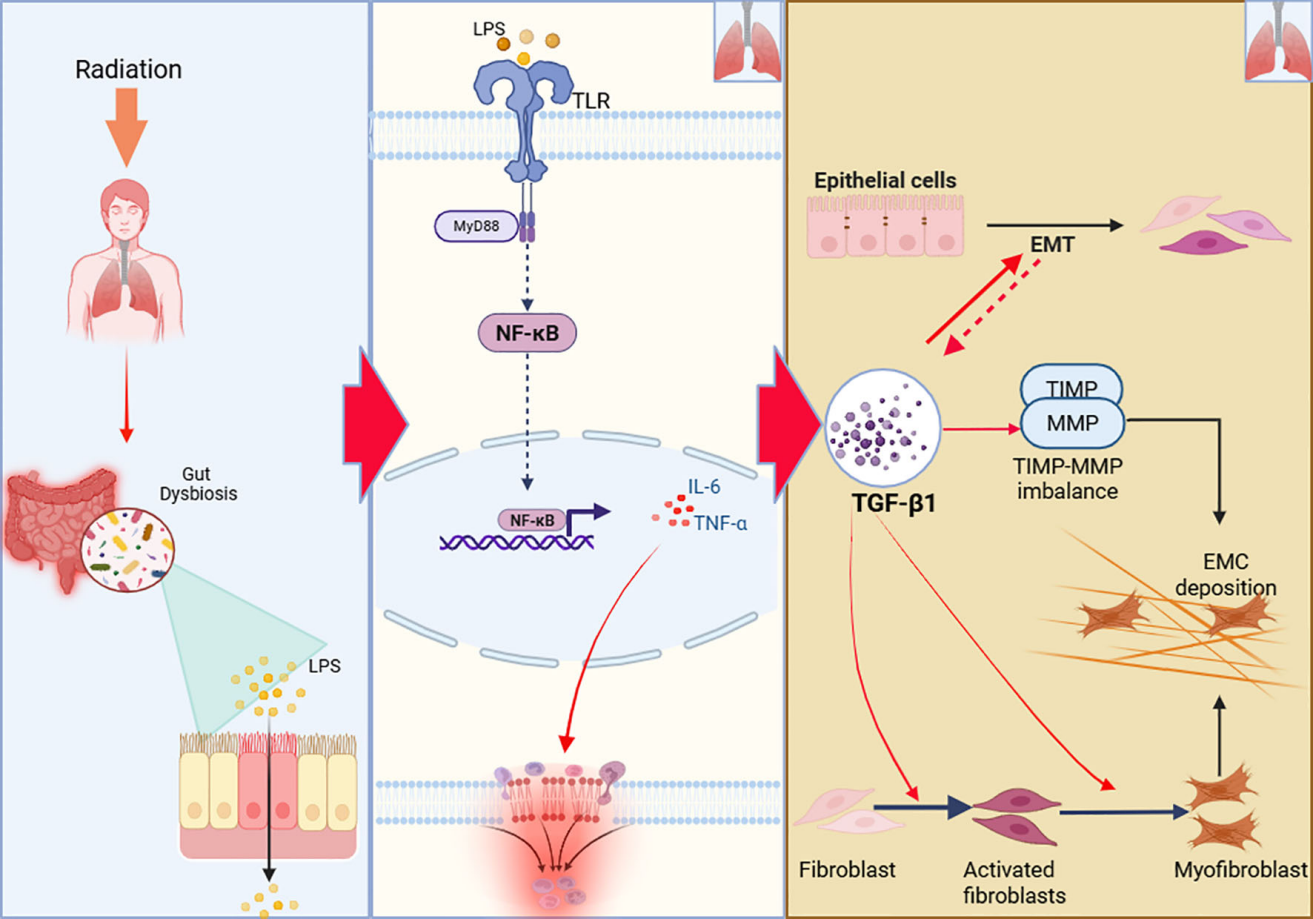
Mechanistic cascade of gut–lung axis disruption in RILI: LPS translocation and inflammation-to-fibrosis transition. The image illustrates the RILI cascade mediated by disruption of the gut–lung axis: ionizing radiation impairs intestinal epithelial integrity through apoptosis induction and suppression of tight junction proteins (occludin and claudin-1), allowing LPS from dysbiotic gut microbiota to translocate systemically via the portal vein. In the lungs, LPS engages TLR4 on alveolar macrophages, recruiting MyD88 to activate NF-κB, which drives the release of proinflammatory cytokines (TNF-α and IL-6) and amplifies epithelial permeability alongside leukocyte influx. This elevation in transforming growth factor-β1 (TGF-β1) promotes EMT in alveolar type II cells, myofibroblast differentiation, and extracellular matrix accumulation (type I/III collagens) via TIMP–MMP imbalance, ultimately resulting in pulmonary fibrosis. Synergistic damage-associated molecular patterns, such as high-mobility group box 1 (HMGB1) from irradiated cells, enhance TLR4 signaling and intensify the shift from inflammation to fibrosis. This conceptual model underscores the potential of gut microbiota-targeted interventions, including TLR4 inhibitors or gut barrier stabilizers, to mitigate RILI progression. RILI, radiation-induced lung injury; LPS, lipopolysaccharide; EMT, epithelial–mesenchymal transition; TIMP, tissue inhibitor of metalloproteinases; MMP, matrix metalloproteinase.

Radiotherapy induces apoptosis in epithelial cells and suppresses proteins such as occludin and claudin-1, thus impairing barrier integrity (38). This direct radiation-induced epithelial damage has been demonstrated in appropriate preclinical animal models of radiotherapy, including fractionated thoracic irradiation in C57BL/6 mice (typically 15-Gy chest RT or clinically relevant fractionated schedules mimicking human thoracic radiotherapy for NSCLC), leading to reduced tight junction integrity and increased gut permeability (34). This change enables pathogenic bacteria and LPS to travel to the lungs via the bloodstream, as confirmed by higher serum LPS levels in these experimental models (39). In contrast, clinical observations in human NSCLC cohorts (N = 45–89) undergoing CRT show similar barrier dysfunction and dysbiosis patterns, but these are primarily associative and often amplified by indirect effects of concurrent chemotherapy, antibiotics, and immunotherapy rather than radiotherapy alone (21). LPS engages TLR4 on alveolar macrophages, triggering the MyD88/NF-κB pathway and elevating proinflammatory cytokines such as TNF-α and IL-6, which foster immune cell influx and vascular permeability. These events advance RILI from acute to chronic phases, as TGF-β drives epithelial–mesenchymal transition (EMT) and fibroblast recruitment. Cytokines from alveolar macrophages draw leukocytes; TGF-β shifts fibroblasts to myofibroblasts, boosting the production of collagen types I and III through imbalances in matrix metalloproteinases (MMPs) and their tissue inhibitors (TIMPs). Notably, TGF-β1 activates fibrotic genes via Smad2/3 signaling (40, 41) (**Figure 2**).

Immune pathways involve intricate cytokine networks. Microbial elements activate DCs via TLRs, aiding ILC2 migration. Tregs curb overactivation, while Th17 cells bolster barriers through IL-17 and IL-22. In RP, radiation frees pathogen-associated molecular patterns, triggering dendritic cell and ILC2 activation alongside Th17-driven fibrosis (32, 42). LPS effects vary by dose: low levels train innate immunity through TLR4/MyD88 for repair, but high levels heighten NLRP3 inflammasome activity and TGF-β-linked fibrosis, worsening RILI (26, 43). In addition to TLR4/NF-κB and TGF-β/Smad routes, gut dysbiosis drives disease progression through SCFA loss (shifting to M1 macrophages), nuclear factor erythroid 2-related factor 2 (Nrf2) suppression, and S1P–S1PR dysregulation (affecting permeability and migration), mainly indicated by preclinical work (20, 21). Radiation alters gut mycobiome diversity, possibly via EV-mediated shifts that trigger NLRP3 inflammasomes and RP-to-RPF progression, with budding parallels in lung mycobiome. Virome imbalances weaken phage bacterial control, promoting Proteobacteria growth, TGF-β signaling, and fibrosis (7, 27). Exosomal miRNAs such as miR-21 and miR-29 modulate redox and fibrotic genes, suggesting their therapeutic potential (44), although supporting clinical evidence is sparse. MiRNA therapies need Phase I trials to gauge their safety and feasibility in radiotherapy settings.

## 4 Gut–lung axis-mediated pathophysiological mechanisms

### 4.1 Metabolite-driven inflammation and fibrosis

Radiotherapy breaches intestinal barriers and triggers gut dysbiosis, shifting metabolite flow to the lungs and remotely shaping immune and tissue responses. The gut–lung axis amplifies RILI through epithelial damage, fibroblast activation, and matrix buildup (45, 46).

#### 4.1.1 SCFAs: dual roles in inflammation and fibrosis

SCFAs reshape immunity via FFAR2/3 (GPR41/43), GPR109A, and HDAC inhibition in animal models (47). In thoracic irradiation mouse models, they dampen neutrophil inflammation and inflammasomes while boosting Tregs and M2 macrophage polarization, thereby attenuating the RP-to-fibrosis transition (48). Human studies have provided partial backing, with RCTs yielding evidence that remains limited due to heterogeneity (I^2^ > 50%) and confounders such as diet or antibiotics (35). At epithelial sites, SCFAs fortify tight junctions, mucins, and Nrf2, mitigating damage in mice (26), yet human variation and species gaps (e.g., Firmicutes/Bacillota dominance in mice vs. Bacteroidetes/Bacteroidota in humans) hinder translation (25). Preclinical hints suggest that low levels of SCFAs engage GPR41/43 on macrophages or DCs, prompting the release of interleukin 10 (IL-10)/TGF-β for Treg support and curbing IL-6/TNF-α. High levels trigger the overactivation of FFAR2 on neutrophils, driving chemotaxis and damage (47), following U-shaped dose-response links observed in cohorts with chronic obstructive pulmonary disease and RILI (limited by biases) (35, 49). Thresholds differ (e.g., butyrate 2–4 mM anti-inflammatory and >10 mM pro-chemotactic) by age, diet, or microbiota (47), calling for optimized delivery (e.g., enteric-coated or lung-targeted), perhaps paired with DAT for protection, as in 2025 acute respiratory distress syndrome models combining SCFA and PGF2α (21, 29). Future work must investigate species differences to distinguish these exploratory links from established paths such as TLR4/NF-κB.

#### 4.1.2 DAT: microbiota-derived antioxidant/anti-fibrotic potential

DAT, produced by *C. orbiscindens* through the metabolism of flavonoids such as quercetin, activates IFN-I signaling in the lung via the phagocyte-dependent induction of interferon-stimulated genes (ISGs) such as IFIT1 and MX1, thereby reducing apoptosis and cytokine storms while preserving pathogen clearance (50). Although direct RILI-specific models remain limited, colonization with *C. orbiscindens* or DAT supplementation replicated these protective effects in viral lung injury models, suggesting translational potential for radiation contexts (50, 51). IFN-Is are closely intertwined with redox and repair processes, tempering ROS levels, aiding alveolar type II cell renewal, dampening TGF-β/Smad by reducing damage-associated molecular patterns, and shifting macrophages toward an anti-inflammatory state. In RILI, subacute DAT may halt pneumonitis-to-fibrosis transition through gut–lung modulation, but human trials are needed to bridge species gaps and differentiate these initial ties from core metabolite paths such as SCFA-GPR41/43 (**Table 2**).

**Table 2:** Key metabolites and exosomal miRNAs in the gut–lung axis of RILI.

Component	Mechanism	Role in RILI	Therapeutic potential
SCFAs (e.g., butyrate and propionate)	Produced by gut microbiota (e.g., Bacteroidetes/Bacteroidota and Firmicutes/Bacillota) via fiber fermentation (26); activate GPR41/43 and inhibit HDACs (47, 52); promote M2 macrophage polarization, Treg differentiation, and NF-κB suppression (33, 52).	Anti-inflammatory effects reduce TNF-α and IL-6 levels (52); alleviate oxidative stress and barrier disruption in radiation-induced dysbiosis (26); dual role—at low levels, mitigate inflammation, and at high levels, may exacerbate fibrosis (47); protect against progression from acute RP to chronic RPF (29, 35).	Supplementation (e.g., dietary fiber or probiotics) to restore gut homeostasis (33); potential in phase-targeted interventions such as intra-radiotherapy administration to reduce RILI severity (20); supported by meta-analyses showing efficacy in ALI models (35).
DAT	Derived from *Clostridium orbiscindens* metabolism of flavonoids (e.g., quercetin) (50); activates type IFN-I signaling via phagocyte-dependent ISGs (e.g., IFIT1 and MX1) (50, 53, 54); synergizes with redox and epithelial repair pathways (30, 31).	Anti-fibrotic and cytoprotective (30, 31); reduces apoptosis, cytokine storms, and ROS (50, 53, 54); modulates gut–lung crosstalk to block pneumonitis-to-fibrosis transition (50, 53, 54); attenuates mucosal inflammation in an IFN-I-dependent manner (50).	Subacute modulation to protect against radiation damage (50, 53, 54); promotes intestinal epithelium regeneration; potential in combination therapies for graft-versus-host disease (GVHD) and cancer immunotherapy (53, 54); requires human studies for species-specific translation (30).
MiR-21 (exosomal miRNA)	Delivered via EVs (55–58); suppresses SMAD7/PTEN, amplifying TGF-β/SMAD2/3 signaling; forms feedback loops promoting EMT and myofibroblast activation (55–58).	Profibrotic (55) mediates fibrogenic activation of pulmonary fibroblasts (55); elevates in recurrent tumors and fibrotic phases, intensifying the inflammation-to-fibrosis shift in RILI (44, 58).	Diagnostic/prognostic biomarker for pulmonary fibrosis progression (58); potential target for neutralizers or inhibitors to halt EMT (56); exosomal delivery in engineered EVs for precision therapy (56, 57).
MiR-29 (exosomal miRNA)	EV-mediated transport (58) regulates redox/fibrosis genes (44); suppresses profibrotic pathways, including TGF-β signaling (58).	Anti-fibrotic (58); inhibits extracellular matrix deposition and fibroblast activation (58); reduces severity of radiation-induced pulmonary changes (44).	Therapeutic agent to suppress fibrosis (58); potential in miRNA-based therapies for RPF (58); serves as a biomarker for disease monitoring in lung diseases (44).
MiR-486-5p (exosomal miRNA)	Engineered in MSC-derived EVs (13, 59); targets GPX4/ACSL4 to inhibit ferroptosis (59); reverses EMT via AKT/GSK3β inhibition (60).	Protective against oxidative stress and cell death (13); alleviates RILI by reducing ferroptosis and fibrosis (59).	May represent a candidate for engineered EV-based approaches for radiation injury treatment (13, 59); suppresses long-term pulmonary fibrosis (59); diagnostic marker in NSCLC and radiation contexts (13); phase I trials needed for safety.

RILI, radiation-induced lung injury; SCFAs, short-chain fatty acids; HDAC, histone deacetylases; RP, radiation pneumonitis; RPF, radiation-induced pulmonary fibrosis; ALI, acute lung injury; DAT, desaminotyrosine; ISGs, interferon-stimulated genes; ROS, reactive oxygen species; EVs, extracellular vesicles; EMT, epithelial–mesenchymal transition; MSC, mesenchymal stem cell.

SCFAs and DAT work in tandem therapeutically: SCFAs aid immune resolution and barrier upkeep through G protein-coupled receptor activation and epigenetic changes, while DAT provides interferon-focused cell protection and redox balance to slow down fibrosis. Clinical translation of therapeutics requires tight control over dose, timing, and delivery, integrated with multi-omics patient grouping to maximize outcomes and safety.

### 4.2 Immune cell cascade responses

#### 4.2.1 Role of gut-derived ILC2 in pulmonary inflammation

Gut-derived ILC2s are fundamental to the gut–lung axis, with priming directed by microbial metabolites. SCFAs from *Bacteroides* and Firmicutes/Bacillota curb ILC2 growth and cytokines (IL-5 and IL-13) via HDAC inhibition and signaling through G protein-coupled receptors, limiting eosinophil influx and remodeling—as observed in airway injury models relevant to radiotherapy (61–64). ILC2 migration hinges on the IL-25/S1P axis (see **Figure 1** for schematic illustration): IL-25 activates ILC2s via IL-17RB, upregulating CCR9 and S1PR1 for S1P-directed lung homing in mouse studies (42). This multistep trafficking is further refined by recent evidence in inflammatory ILC2s (iILC2s): IL-25 induces epigenetic landscape changes with transcription factors KLF2 and ZEB2 driving the upregulation of S1PR1 and S1PR5, respectively; S1PR5 regulates iILC2 migration from the intestine into the lymph, whereas S1PR1 controls iILC2 egress from mesenteric lymph nodes to blood and then to distal tissues including the lung, where redistributed ILC2s contribute to tissue repair (65). **Figure 1** also depicts potential activation of circulating ILC2 by translocated LPS; this mechanism is supported by preclinical *in vivo* studies demonstrating direct TLR4-mediated activation of ILC2s by LPS, leading to enhanced type 2 cytokine production and eosinophilic airway inflammation, although such direct ILC2 activation by LPS has not yet been specifically demonstrated in radiotherapy-induced lung injury models and remains associative in the RILI context via gut dysbiosis and barrier breach (42, 65). Once in the lungs, ILC2s release IL-5, IL-13, and amphiregulin, aiding tissue repair but triggering fibrosis via IL-33/ST2, TGF-β boosts, and collagen buildup (66). Metabolic ties include SCFA-downregulated shifts and amino acid-fueled mTORC1–oxidative phosphorylation (67–70). S1P blockers such as FTY720 reduce immune cell influx in models, hinting at targets despite radiotherapy infection risks. High-fiber diets or FMT can alleviate RILI by restoring gut microbes, although human RCTs are needed due to species differences.

#### 4.2.2 Treg/Th17 imbalance and its association with pulmonary fibrosis

Intestinal metabolites modulate Treg/Th17 balance differently. SCFAs foster Treg formation and stability through GPR43/HDAC inhibition, while commensals and proinflammatory settings (IL-6/IL-1β) drive Th17 skewing. Post-radiotherapy gut dysbiosis and a compromised barrier reduce SCFA production and exacerbate microbe- or damage-associated patterns, shifting toward Th17 dominance and Treg loss and subsequently raising IL-17A/granulocyte–macrophage colony-stimulating factor tensions and fibrosis risk (71, 72). During disease progression, Th17/IL-17A triggers fibrosis through neutrophil pull/neutrophil extracellular trap formation, STAT3–TGF-β fibroblast synergy, and a rise in collagen/α-smooth muscle actin. Tregs dampen inflammation and fibroblast activity via IL-10/TGF-β. RILI phases—from acute (IL-1β/IL-6/TNF-α/IL-17A spikes) to chronic (type 2/TGF-β-led remodeling)—hinge on gut–lung metabolic-immune links, prompting resolution or worsening (73–75).

### 4.3 EVs and pathway networks

RILI progresses from acute inflammation to chronic fibrosis, affecting 10% to 30% of thoracic radiotherapy patients with RP or RPF (43, 76). The gut–lung axis drives this progression through EVs released from disrupted intestinal barriers and gut microbiota. These EVs carry miRNAs, proteins, sphingolipids, and nucleic acids that remotely modulate lung immune responses (77, 78). In the acute inflammatory phase, gut-derived EVs containing LPS and regulatory miRNAs activate the TLR4-MyD88/NF-κB pathway in alveolar macrophages, elevating IL-6, TNF-α, and CCL2 levels while promoting neutrophil recruitment (79). This established mechanism, confirmed in preclinical LPS challenge and thoracic irradiation models, can be targeted by TLR4 inhibitors or miR-146a-engineered EVs. Concurrently, EV-associated sphingolipids, ATP, and oxidized mitochondrial DNA trigger NLRP3 inflammasome activation, leading to macrophage pyroptosis and further barrier disruption (80).

During the transitional phase, radiotherapy-released nuclear and mitochondrial DNA, together with EV nucleic acids, engages the cGAS–STING pathway to induce type I interferon responses and monocyte/macrophage recruitment (81, 82). Gut-derived prostaglandin F2α (PGF2α), upregulated by FMT, further activates MAPK/NF-κB signaling to scavenge ROS and limit apoptosis in a context- and dose-dependent manner. Circulating S1P interacts with S1PR1/3 and EV sphingolipids to activate Rho/ROCK, destabilizing VE-cadherin junctions and increasing vascular permeability, causing edema (81, 83, 84).

In the chronic fibrotic phase, EVs deliver profibrotic miRNAs such as miR-21, which suppress SMAD7/PTEN to amplify TGF-β/SMAD2/3 signaling, EMT, and myofibroblast differentiation (55–59). Recent preclinical advances show that mesenchymal stem cell (MSC)-derived EVs engineered with miR-486-5p inhibit ferroptosis via GPX4/ACSL4 regulation, thereby reducing oxidative stress and long-term fibrosis in RILI models (59). MSC-EVs may also target the intestine to modulate the gut–lung axis (85).

**Figure 3:**
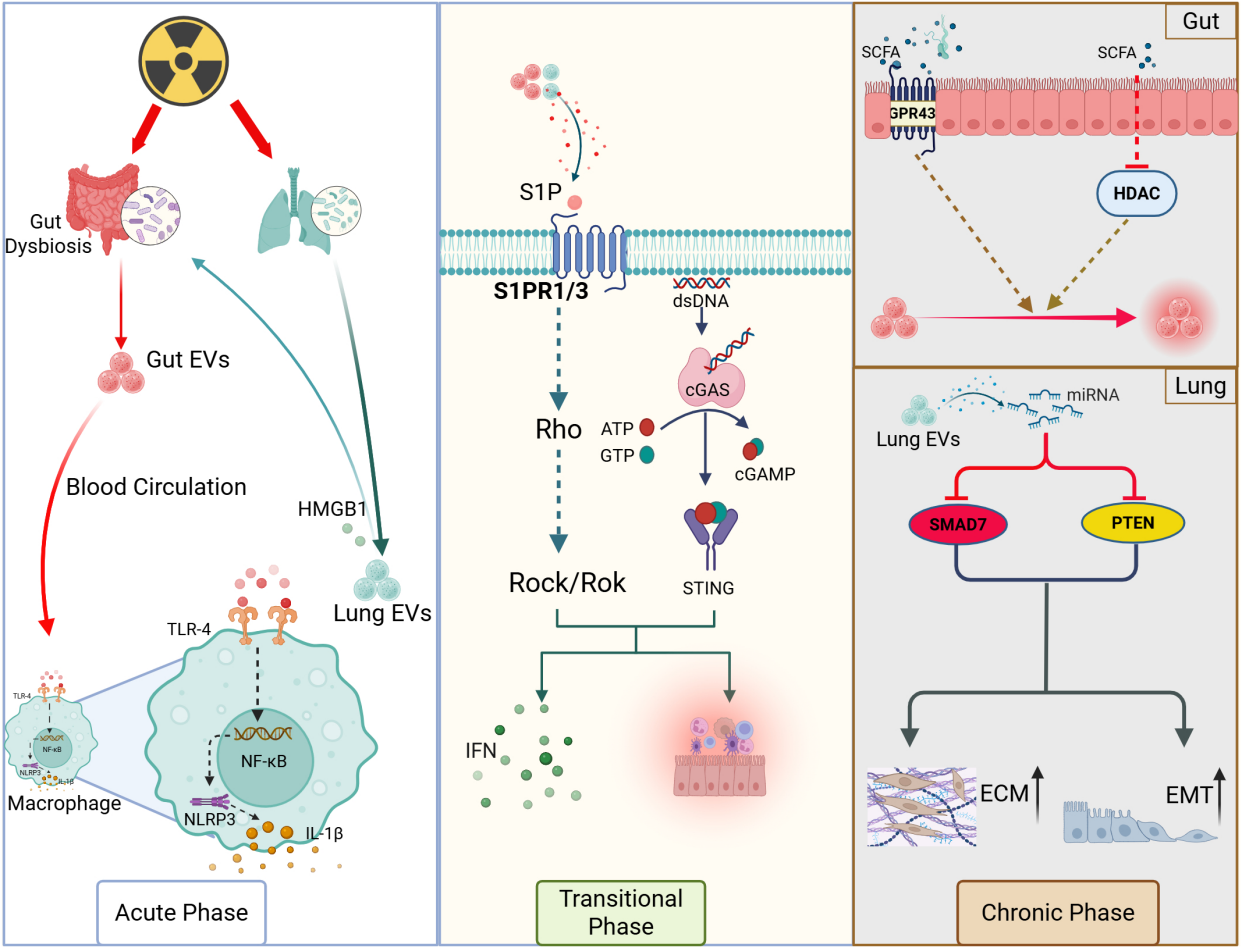
Phased gut–lung axis mechanisms mediated by EVs in RILI. This schematic shows phased gut–lung axis mechanisms in RILI (acute, transitional, and chronic). Radiation triggers gut dysbiosis; gut EVs circulate to activate lung EVs/macrophages. Acute phase: TLR4 detects patterns (e.g., HMGB1), activating NF-κB/NLRP3 for IL-1β/IFN release and inflammation. Transitional: S1P/S1PR1/3 and cGAS–STING amplify IFN, shifting to fibrosis. Chronic: SCFA depletion shifts GPR43/HDAC proinflammatory; lung EVs with miR-21 inhibit PTEN/SMAD7, promoting EMT/ECM deposition and fibrosis. Mechanisms rely on metabolites, migration, and EV communication, per animal models; translation addresses species differences/antibiotic confounders. EVs, extracellular vesicles; RILI, radiation-induced lung injury; EMT, epithelial–mesenchymal transition; ECM, extracellular matrix.

Among these EV-mediated processes, miRNA profiling (e.g., miR-486-5p and miR-21) represents the most actionable biomarker and therapeutic target because of its direct links to fibrosis and the feasibility of engineered delivery. Declines in SCFAs further shift EV cargo toward proinflammatory profiles, exacerbating TLR4 and NLRP3 responses (86). These mechanisms are predominantly established in preclinical models; human translation requires caution due to species differences and confounders. Blended strategies such as fiber supplementation, SCFA-producing transplants, and EV-omics tracking have been investigated for potential alleviation of RP and improvement in radiotherapy tolerance, with 2025–2026 insights from Li, Huang, Pu, and Wang emphasizing the need for human trials (13, 87, 88) (**Figure 3**).

## 5 Clinical evidence and intervention strategies

Clinical and preclinical data link radiotherapy to gut microbiome shifts—lower α-diversity, SCFA output, and pathogen growth—associated with the onset of RP. The gut–lung axis exacerbates RILI, paving the way for microbiome biomarkers and tailored interventions. This part ties evidence to practical designs, combining dysbiosis detection, treatments, and therapies with dosimetry, radiomics, and multi-omics for clinical application.

### 5.1 Microbiota monitoring before and after RT and the association between declining intestinal microbiota diversity and RP risk

Radiotherapy induces gut microbiome alterations, including reduced α-diversity, SCFA depletion, and pathogen growth, which are linked to RP primarily identified through observational studies (89). In thoracic cancers such as NSCLC, these changes—particularly reduced gut microbiota stability—correlate with increased RP risk. A key longitudinal NSCLC cohort (N = 52) demonstrated that lower intra-patient gut microbiota stability during CRT, measured using 16S rRNA sequencing, was independently associated with a higher incidence of grade ≥2 RP in multivariable-adjusted models (p < 0.05), accompanied by a 25% decrease in α-diversity and the proliferation of Proteobacteria; baseline *Faecalibacterium* abundance has protective properties (15). Non-invasive markers such as 16S rRNA sequencing and serum metabolomics, guided by preclinical LPS translocation and SCFA loss pathways, may enable early risk stratification and personalized care.

However, these associations remain correlative and are heavily confounded by antibiotics, concurrent chemotherapy, and immunotherapy, which independently disrupt the gut microbiota and mimic or exacerbate RILI (observational evidence is limited by the high risk of bias, inconsistency with I^2^ > 50%, and imprecision from small sample sizes of N = 45–89) (34). Species differences between mice (Firmicutes-dominant) and humans (Bacteroidetes-dominant) further limit translation, and negative or null findings in antibiotic-exposed subgroups underscore the need for layered analyses and Mendelian randomization to establish causality. Gut microbiota profiles thus serve as promising early biomarkers, but large-scale, stratified RCTs are required to validate their utility alongside AI-integrated multi-omics and radiomics for RP prediction. Proton therapy reduces gut irradiation, which may preserve microbiota stability and synergize with monitoring strategies, potentially lowering RILI incidence by 15%–20% in thoracic cohorts (**Figure 2**).

### 5.2 Prebiotic/probiotic interventions

Prebiotics and probiotics restore gut microbiota composition and metabolite production, thereby strengthening the gut–lung axis and attenuating RILI. In preclinical thoracic irradiation models, strains such as *Lactobacillus acidophilus* and *B. longum* reduce TNF-α and IL-6 levels, preserve epithelial barrier integrity, and improve lung function parameters (89). Pilot clinical trials have indicated that these interventions reverse radiotherapy-induced gut damage, promote crypt/villus regeneration, and support pulmonary outcomes when combined with prebiotic fibers such as cellulose for optimized colonization in high-risk patients. SCFA supplementation further enhances these effects through GPR41/43 and HDAC inhibition, promoting Treg differentiation and M2 macrophage polarization. Although promising, current evidence is primarily preclinical with limited pilot data; well-designed RCTs with targeted delivery (e.g., enteric-coated or nanoparticle formulations) are essential to confirm efficacy and optimal dosing.

### 5.3 FMT and special metabolites

FMT and targeted metabolites represent advanced microbiome-based options for RILI. Preclinical mouse thoracic irradiation studies demonstrate that FMT reshapes gut metabolic homeostasis, elevates protective metabolites such as prostaglandin F2α (PGF2α), activates MAPK/NF-κB signaling, and triggers FAM134B-dependent ER-phagy, thereby reducing RP severity, oxidative stress, lung wet/dry ratios, and fibrosis (20, 87). Oncology trials link FMT to symptom relief, suggesting benefits for chronic cases of RP (17, 20). Early clinical pilot studies and oncology trials have reported improvements in lung function and inflammation relief, with a 2025 single-arm study confirming the safety of interventions for radiation-related gastrointestinal toxicity, which may extend to pulmonary therapy applications (18, 19, 89). However, human RILI-specific data remain limited by donor variability and the absence of large RCTs. Complementary approaches include microbiota-derived metabolites such as DAT (which has been shown in preclinical models to enhance type I interferon signaling and reduce radiation-induced apoptosis and cytokine storms) (51) and SCFAs (which support immune balance and barrier function) (90). Small-molecule agents, including berberine, can enrich beneficial taxa such as *Akkermansia muciniphila* and elevate microbiota-derived inosine, modulating RILI via the gut–lung axis in a microbiota-dependent manner (91). These oral agents may address some FMT limitations and have been investigated for potential interactions with immunotherapy through the modulation of CD8+ T-cell responses and the mitigation of immune-related adverse events. FMT has been among the most extensively studied interventions in preclinical models for metabolic remodeling, while small molecules offer a more accessible translational approach; both require stratified RCTs to establish clinical benefit.

## 6 Discussion and future perspectives

Evidence for the gut–lung axis in RILI remains limited by predominantly observational study designs, small sample sizes (often N < 89), and multiple confounders including antibiotics, chemotherapy, immunotherapy, smoking status, and comorbidities (35, 49). In NSCLC cohorts, gut microbiota instability is associated with over 30% higher RP risk (15), yet these findings lack randomization and are weakened by unadjusted confounders. Recent comprehensive reviews have provided valuable microbial perspectives on radiation-induced injury and the gut microbiota (107). Overall evidence from observational studies is limited by the high risk of bias, inconsistency (I^2^ > 50%), and positive reporting bias; recent longitudinal cohorts have begun to strengthen associative links, but large, well-controlled RCTs are urgently needed (**Table 1**). Preclinical mechanistic insights (e.g., TLR4/NF-κB, SCFA-GPR41/43, cGAS–STING, ILC2 trafficking, Treg/Th17 imbalance, and EV signaling) are robust, yet mouse–human microbiota differences and selective reporting limit direct translation (36–41, 71).

### 6.1 Dynamic Metabolite-Threshold Framework: elaboration and clinical integration

**Figure 4:**
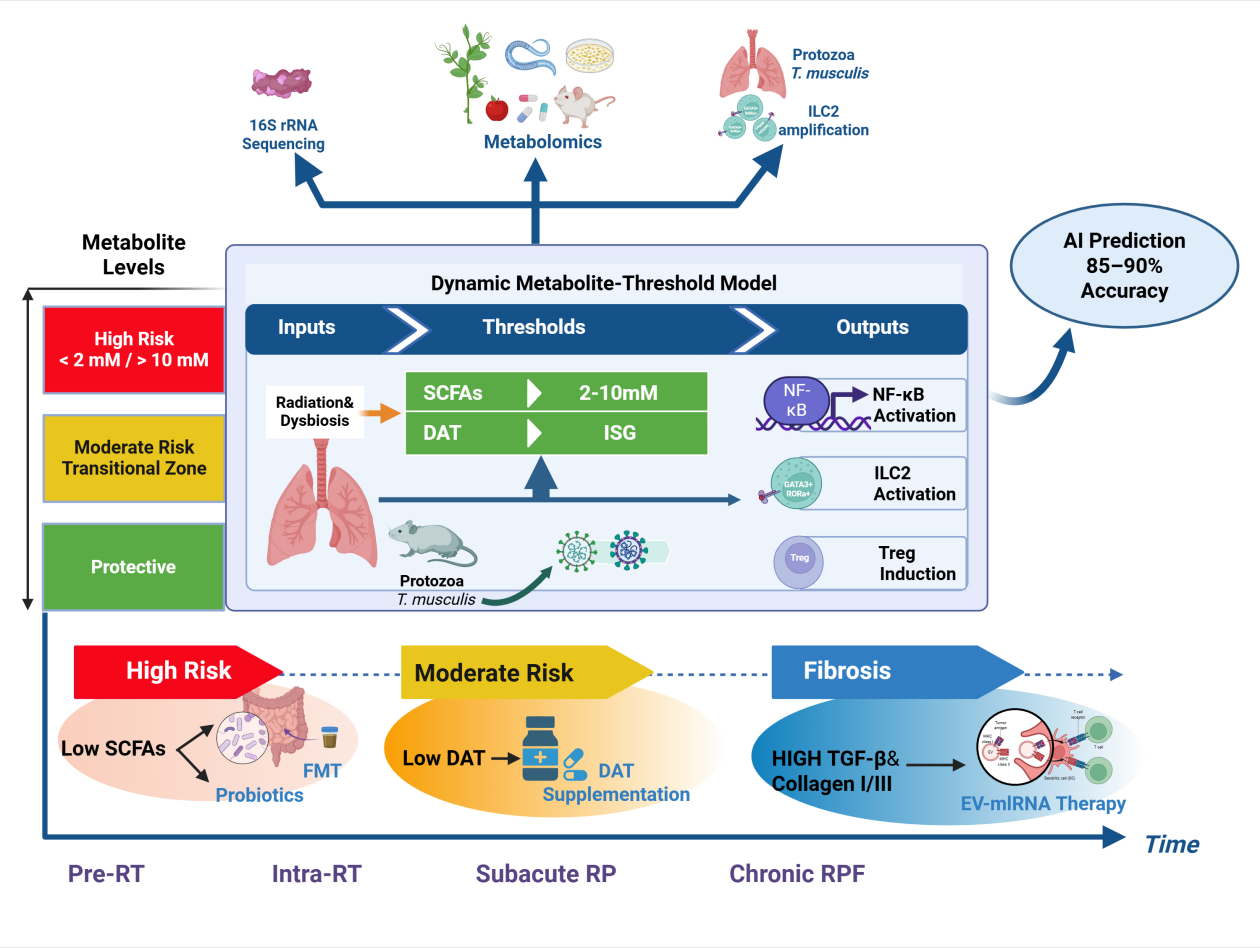
Dynamic metabolite-threshold framework for gut–lung axis in RILI. An ODE-based model shows thoracic radiation and gut dysbiosis driving SCFA (protective at 2–4 mM vs. profibrotic >10 mM) and DAT thresholds, determining immune outcomes (TLR4/NF-κB activation vs. Treg induction). The x-axis depicts temporal RILI phases; the y-axis shows metabolite concentrations with color-coded risk zones (green, protective; yellow, transitional; red, pathogenic). Includes multi-omics biomarkers, AI-based risk prediction (85%–90% accuracy), protozoal influences on ILC2, and phase-specific interventions (FMT for severe SCFA depletion; DAT/SCFA supplementation for moderate depletion). RILI, radiation-induced lung injury; SCFA, short-chain fatty acid; DAT, desaminotyrosine; FMT, fecal microbiota transplantation.

As a hypothesis-generating conceptual model, the Dynamic Metabolite-Threshold Framework (DMTF) integrates radiotherapy-induced shifts in SCFAs, DAT, and immune thresholds to explore disease progression and the potential timing of interventions in the gut–lung axis of RILI. Building on observed U-shaped SCFA dose responses (anti-inflammatory at 2–4 mM but potentially chemotactic at >10 mM) and DAT-mediated type I interferon protection reported in preclinical models (47, 50), the framework uses illustrative ordinary differential equations and AI-supported multi-omics integration (16S rRNA, metabolomics, radiomics, and dosiomics) for exploratory RP risk forecasting. Preliminary AI models have reported approximately 75% accuracy (92). The application of the DMTF to clinical decision-making can be explored through pre-radiotherapy multi-omics screening to identify patients with low baseline SCFAs/DAT, intra-radiotherapy SCFA supplementation to sustain GPR41/43 signaling, subacute-phase DAT modulation, and EV-miRNA-guided MSC-EV therapy for established RPF (e.g., miR-486-5p). In NSCLC cohorts, such an exploratory framework may aid risk stratification alongside interventions such as FMT or proton therapy (93); however, any potential reductions in RP incidence require validation in prospective, stratified RCTs. Multi-kingdom contributions, including protozoa, are addressed in the Future Perspectives section (**Figure 4**).

#### 6.1.1 Validation and growth

To fill RCT voids, multi-arm trials are suggested. Example: Phase II/III RCT (N = 300–00) in RT-treated NSCLC patients, stratified by recent antibiotic use, RT type (proton vs. photon), and Immune Checkpoint Inhibitors (ICIs). Arms: FMT capsules weekly, SCFA daily (enteric butyrate/propionate), placebo, optional FMT + SCFA. Main endpoint: grade 2+ RP at 6 months; secondary: FEV1 shift, microbiota changes. Power: 80% for 20%–30% reduction. Pilot RCTs (N > 200, antibiotic-layered) test the DMTF, adding protozoal sequencing for kingdom thresholds (94). Expansions: CRISPR-FMT for metabolite targeting, ODE-AI for live apps. Backing: 2025 AI (75% accuracy) (95), multi-omics spotting radioprotectors; post-2025 RCTs validate with randomization. Meta-analyses (Xie et al, 2024) show that SCFAs dose-dependently reduce the lung wet/dry ratio (Standardized Mean Difference (SMD) −2.75) in acute lung injury (ALI) models, hinting at barriers in RILI.

### 6.2 Phase-specific strategies and emerging therapies

Building upon the DMTF in Section 6.1 and illustrated in **Figure 4**, we propose phase-specific strategies to translate gut–lung axis insights into clinical practice for RILI. Pre-radiotherapy multi-omics screening of microbiota and metabolites (e.g., SCFAs and DAT) enables risk stratification and baseline optimization. During radiotherapy, SCFA supplementation via dietary fiber, probiotics, or enteric formulations sustains GPR41/43 signaling and Treg/M2 polarization to mitigate acute RP (preclinical and emerging 2025–2026 pilot data) (96, 97). In the subacute phase after RP onset, DAT modulation through *C. orbiscindens* enrichment or flavonoid supplementation harnesses IFN-I pathways to limit progression to RPF. For established RPF, engineered MSC-derived EVs carrying miR-486-5p provide targeted antifibrotic effects via ferroptosis inhibition and EMT reversal. These approaches are enhanced by proton therapy’s microbiota-sparing profile, which reduces off-target gut irradiation compared with photon therapy and may synergize with the SCFA modulation of anti-PD-1 immunotherapy responses (98, 99).

Major gaps persist in the RCT landscape: small pilot studies lack stratification for confounders (antibiotics, chemotherapy, and immunotherapy) and head-to-head comparisons of FMT with SCFA versus EV therapies. For post-RT RP patients, we recommend adaptive multi-arm Phase II/III RCTs (target N = 400), with cohorts stratified by antibiotic exposure, baseline dysbiosis, and dosimetric risk (V20 > 20%). Trial arms can include FMT capsules, daily SCFA supplementation, miR-486-5p-engineered EV therapy, and placebo, with primary endpoints of grade ≥2 RP incidence, High-Resolution Computed Tomography (HRCT) fibrosis scores, and secondary measures including Treg/Th17 balance, EV-miRNA profiles, and FEV1 improvement. Such designs would elevate evidence quality beyond current Grading of Recommendations Assessment, Development and Evaluation (GRADE) low-to-moderate levels (17, 35, 95).

#### 6.2.1 Impact of immunotherapy on the gut–lung axis in irradiated patients

ICIs such as anti-PD-1/PD-L1 enhance antitumor immunity, but when combined with radiotherapy, they increase RILI risk through immune-related adverse events such as ICI pneumonitis mimicking RP (100). Gut dysbiosis modulates ICI efficacy and toxicity via metabolites and immune cell trafficking: beneficial taxa (*Bifidobacterium*, *Faecalibacterium*) promote CD8+ T-cell and IFN-γ responses, while the proliferation of Proteobacteria and depletion of SCFA amplify TLR4/NF-κB-driven inflammation and Treg/Th17 imbalance in RT-ICI NSCLC cohorts (101, 102). Pre-ICI microbiota screening, probiotic/SCFA supplementation, or FMT may mitigate immune-related adverse events (irAEs) and enhance outcomes, but dedicated stratified RCTs (N = 300) comparing FMT with SCFA in RT-ICI patients are needed (GRADE: moderate for RCTs and low for cohorts due to heterogeneity).

#### 6.2.2 Impact of proton therapy on the gut–lung axis

Proton therapy employs the Bragg peak for precise dose delivery and spares normal tissues, resulting in lower off-target gut irradiation compared with photon-based approaches. This may help preserve microbiota diversity, SCFA production, and barrier integrity, potentially limiting LPS translocation, ILC2 migration, and progression from RP to RPF; current evidence is low-to-moderate GRADE from small cohorts. Preclinical models have reported milder dysbiosis and radioprotective metabolite shifts with proton therapy. Multicenter multi-arm RCTs (N > 200) comparing proton with photon therapy, with or without microbiome interventions and stratified by antibiotic exposure, are required to evaluate any microbiota preservation effects and their impact on RILI.

In summary, this review synthesizes evidence on the gut–lung axis in radiation-induced lung injury, encompassing acute RP and chronic radiation-induced pulmonary fibrosis. Radiotherapy disrupts gut ecology (for example, the depletion of *C. orbiscindens*), compromises intestinal barriers, and depletes metabolites, contributing to inflammation and fibrogenesis through pathways such as TLR4/NF-κB, TGF-β/Smad, S1P–S1PR, and cGAS–STING; these mechanisms are causal in rodent models and remain largely correlative in humans, with FMT studied in some RCTs (36–41, 81). SCFAs produced by *Bacteroides* species and DAT produced by *C. orbiscindens* have been investigated for their roles in modulating these responses. SCFAs act via GPR41/43 and HDAC inhibition to promote Treg and M2 macrophage differentiation and downregulate TNF-α and IL-6, exhibiting a U-shaped dose response; DAT stimulates type I IFN signaling and may attenuate ROS, apoptosis, and progression from RP to RPF (50). Additional mechanisms include ILC2 migration via the IL-25/S1P axis, contributing to type 2 responses and extracellular matrix deposition, as well as Treg/Th17 imbalance favoring IL-17A-driven profibrotic effects, as observed in experimental and cohort studies (32, 42, 71, 72). EVs mediate effects via miR-21, miR-486-5p, and nucleic acids that engage NLRP3 and cGAS–STING pathways, with supporting data from animal and limited human studies (77–79, 103, 104). Observational cohorts have linked gut dysbiosis to increased RP risk (OR approximately 3.1); interventions such as probiotics, SCFA supplementation, and FMT have shown effects on microbiota restoration in some RCTs, although challenges include donor variability and interactions with radiotherapy and immunotherapy (17, 105). The review discusses a conceptual model integrating dosimetry and ecological factors while noting limitations such as interspecies microbiota differences (mouse Firmicutes/Bacillota versus human Bacteroidetes/Bacteroidota dominance) and trial design issues. Future multicenter RCTs (N > 200, stratified by antibiotic exposure) are needed to test FMT, proton therapy, and SCFA supplementation in combination with anti-PD-1 therapy, with endpoints including RP incidence and FEV1. Such trials should also evaluate metabolite-threshold AI approaches and novel delivery systems such as CRISPR-engineered FMT capsules. Overall, multi-omics precision strategies may address patient heterogeneity, and adequately powered RCTs remain essential to bridge current evidence gaps (95). Mechanistic aspects are robust, while clinical evidence is limited by sizes/confounders such as antibiotics. Preclinical biases involve murine overstatement of the axis due to differences (human Bacteroidetes/Bacteroidota vs. mouse Firmicutes/Bacillota), failing human variability, and translation gaps (rodent FMT, not human) (34, 106). The literature is subject to publication bias, which favors positive results and marginalizes negative findings (30). While the narrative review format partially mitigates this limitation, the majority of clinical observations are associative rather than causal, unlike the mechanistic insights derived from preclinical models.. Gaps include few trials or large RCTs for FMT/SCFAs/DAT, a limitation compounded by interferences (SCFA-IFN PD-1 and high-dose risks) (98); 2025 reviews underreported the negative outcomes associated with FMT (19). We address these gaps through a detailed discussion of underlying mechanisms, thereby laying the groundwork for future research advances. Networks refine biomarkers (16S rRNA gut dysbiosis and EV-miRNA fibrosis) for stratified precision. Phase II/III RCTs assessed FMT, SCFAs, and DAT, with the endpoint being RP/FEV1/fibrosis scores, controlling for confounders. Post-2025 studies validated 15%–25% RP/10%–20% FEV1 gains. AI metabolite thresholds optimize SCFA/PGF2α, with 20%–30% RP reduction according to 2025 studies (75% accuracy); CRISPR-based smart capsules can help overcome major translational hurdles, such as targeted delivery, microbiota engraftment, and controlled metabolite release. Such approaches can be effectively integrated with dysbiosis-minimizing proton therapy and SCFA supplementation in combination with anti-PD-1 immunotherapy (98). Long-term studies evaluated durability; AI radio-microbiome multi-omics predicted RILI, reducing the incidence of toxicity in NSCLC by 15%–25%. Frameworks for precision oncology provide balanced evidence. Cross-disease comparisons showed that RILI shares implications with chronic obstructive pulmonary disease (COPD), asthma, and COVID-19, establishing it as a distinct acute insult in the broader landscape of respiratory disorders, where it informs strategies for enhancing lung resilience and guiding treatments. Common features include patterns of gut dysbiosis, such as Proteobacteria dominance and SCFA depletion, that amplify LPS/TLR4 signaling in RILI and COPD flares, dampen type 2 immune responses in asthma (43), and promote cytokine surges in COVID-19. Across these conditions, SCFAs support Treg activity and mucosal barriers, whereas disruptions in ILC2, Treg, and Th17 balance promote fibrosis (30, 32); protozoal elements further shape Th2/ILC2 dynamics, worsening outcomes in asthma and COVID-19 in patterns akin to those observed in RILI. In contrast, RILI features an acute, often reversible dysbiosis triggered by radiation (1, 106), differing from the persistent smoking-related Firmicutes/Bacillota proliferation in COPD, allergen-driven Bacteroidetes/Bacteroidota skewing in asthma, and virus-induced mycobiome or IFN changes in COVID-19. RILI’s added layers stem from interactions between radiotherapy and immunotherapy, which are elements not present in these non-oncologic diseases (98); protozoal impacts also diverge, as radiation impairs antiviral mechanisms in ways unlike the adaptive shifts in COPD or viral amplifications in COVID-19. Considering RILI as a model within this continuum, thresholds for SCFAs drawn from asthma and COPD studies can refine interventions, and protozoal roles may be better delineated via ODE models of their metabolite interactions. Such comparisons of dysbiosis, triggers, and therapies highlight the urgency for post-2025 research on overlapping pathways and protozoal contributions. Overall, this integration links RILI to wider respiratory microbiology (**Table 1**).

### 6.3 Future perspectives on multi-kingdom (including protozoal) contributions to the gut–lung axis in RILI

While bacterial metabolites (SCFAs and DAT) and EVs dominate current evidence in RILI, emerging inter-kingdom research highlights protozoa as potential modulators of the gut–lung axis. Burrows et al. demonstrated that the murine gut commensal protozoan *Tritrichomonas musculis* (T.mu) drives IL-25-dependent activation and S1P-receptor-mediated (S1PR1/S1PR5) migration of iILC2s from the intestine to the lung, forming a tripartite network with lung-resident CD4+ T cells and B cells, which promotes perivascular eosinophilia. This exacerbates type 2 allergic airway inflammation while conferring protection against disseminated *Mycobacterium tuberculosis* infection; protozoan DNA was also detected in sputum from patients with severe allergic asthma (5, 94).

In the context of RILI, these findings generate testable hypotheses: radiation-induced gut barrier disruption and dysbiosis can theoretically amplify T.mu-driven ILC2 trafficking, potentially influencing the RP-to-RPF transition via enhanced type 2 cytokine (IL-5/IL-13) and TGF-β signaling—pathways already implicated in RILI fibrosis. Protozoal EVs or nucleic acids may further engage cGAS–STING or NLRP3 in lung macrophages. However, no preclinical RILI models have incorporated protozoal colonization, and human data are lacking. Future studies should employ gnotobiotic mice (T.mu-colonized ± thoracic irradiation), multi-kingdom 16S/18S/ITS sequencing in RILI cohorts, and stratified RCTs assessing protozoal status as a modifier of microbiome interventions (FMT/SCFA). Integrating protozoal profiling into the DMTF can refine risk prediction, particularly in immunotherapy-combined regimens. Such work would address current gaps in multi-kingdom causality while advancing precision microbiome therapies for RILI (5, 94).
